# Effect of a 4-Week Telerehabilitation Program for People With Post-COVID Syndrome on Physical Function and Symptoms: Protocol for a Randomized Controlled Trial

**DOI:** 10.1093/ptj/pzae080

**Published:** 2024-06-29

**Authors:** Jack M Reeves, Lissa M Spencer, Ling-Ling Tsai, Andrew J Baillie, Yuna Han, Regina W M Leung, Joshua A Bishop, Lauren K Troy, Tamera J Corte, Alan K Y Teoh, Matthew Peters, Carly Barton, Lynette Jones, Jennifer A Alison

**Affiliations:** Physiotherapy Department, Royal Prince Alfred Hospital, Sydney, NSW, Australia; Sydney School of Health Sciences, Faculty of Medicine and Health, the University of Sydney, Sydney, NSW, Australia; Physiotherapy Department, Royal Prince Alfred Hospital, Sydney, NSW, Australia; Sydney School of Health Sciences, Faculty of Medicine and Health, the University of Sydney, Sydney, NSW, Australia; Physiotherapy Department, Royal Prince Alfred Hospital, Sydney, NSW, Australia; Sydney School of Health Sciences, Faculty of Medicine and Health, the University of Sydney, Sydney, NSW, Australia; Sydney School of Health Sciences, Faculty of Medicine and Health, the University of Sydney, Sydney, NSW, Australia; Allied Health Professorial Unit, Sydney Local Health District, Sydney, NSW, Australia; Physiotherapy Department, Canterbury Hospital, Sydney, NSW, Australia; Physiotherapy Department, Concord Repatriation General Hospital, Sydney, NSW, Australia; Respiratory Medicine, Concord Repatriation General Hospital, Sydney, NSW, Australia; Physiotherapy Department, Balmain Hospital, Sydney, NSW, Australia; Department of Respiratory and Sleep Medicine, Royal Prince Alfred Hospital, Sydney, NSW, Australia; Sydney Medical School, Faculty of Medicine and Health, the University of Sydney, Sydney, NSW, Australia; Department of Respiratory and Sleep Medicine, Royal Prince Alfred Hospital, Sydney, NSW, Australia; Sydney Medical School, Faculty of Medicine and Health, the University of Sydney, Sydney, NSW, Australia; Department of Respiratory and Sleep Medicine, Royal Prince Alfred Hospital, Sydney, NSW, Australia; Sydney Medical School, Faculty of Medicine and Health, the University of Sydney, Sydney, NSW, Australia; Respiratory Medicine, Concord Repatriation General Hospital, Sydney, NSW, Australia; Sydney Medical School, Faculty of Medicine and Health, the University of Sydney, Sydney, NSW, Australia; Department of Respiratory and Sleep Medicine, Royal Prince Alfred Hospital, Sydney, NSW, Australia; Department of Respiratory and Sleep Medicine, Royal Prince Alfred Hospital, Sydney, NSW, Australia; Sydney School of Health Sciences, Faculty of Medicine and Health, the University of Sydney, Sydney, NSW, Australia; Allied Health Professorial Unit, Sydney Local Health District, Sydney, NSW, Australia

**Keywords:** Community-Acquired Infections, Physical Therapists, Pulmonary Rehabilitation, Respiratory System, Telemedicine

## Abstract

**Objective:**

COVID-19 has led to significant morbidity and mortality globally. Post-COVID sequelae can persist beyond the acute and subacute phases of infection, often termed post-COVID syndrome (PCS). There is limited evidence on the appropriate rehabilitation for people with PCS. The aim of this study is to evaluate the effect on exercise capacity, symptoms, cognition, anxiety, depression, health-related quality of life, and fatigue of a 4-week, twice-weekly supervised pulmonary telerehabilitation program compared with usual medical care for people with PCS with persistent respiratory symptoms.

**Methods:**

The study will be a multi-site randomized controlled trial with assessor blinding. Participants with confirmed previous COVID-19 infection and persistent respiratory symptoms who attend a post-COVID respiratory clinic will be randomized 1:1 to either an intervention group of 4 weeks, twice-weekly pulmonary telerehabilitation or a control group of usual medical care. Participants in the control group will be invited to cross-over into the intervention group after the week 4 assessment. Primary outcome: exercise capacity measured by the 1-minute sit-to-stand test. Secondary outcomes: 5 repetition sit-to-stand test; Montreal Cognitive Assessment; COVID-19 Yorkshire Rehabilitation Scale; Chronic Obstructive Pulmonary Disease Assessment Test; 36-Item Short-Form Health Survey; Hospital Anxiety and Depression Scale; Fatigue Severity Scale; and the Kessler Psychological Distress Scale. Outcomes will be collected at baseline, after 4-weeks intervention or control period, after intervention in the cross-over group, and at 12-month follow-up.

**Impact:**

Research into effective rehabilitation programs is crucial given the substantial morbidity associated with PCS and the lack of long-term data for COVID-19 recovery. A short-duration pulmonary telerehabilitation program, if effective compared with usual care, could inform practice guidelines and direct future clinical trials for the benefit of individuals with persistent respiratory symptoms post-COVID.

## Introduction

The COVID-19 pandemic has led to significant global morbidity and mortality.^1^ In Australia, there have been more than 11 million recorded COVID-19 infections, with over 19,000 directly attributable deaths.^2^ Persistent symptoms post-COVID, commonly termed post-COVID syndrome (PCS), Long COVID, or post-acute sequelae of COVID, can lead to a considerable negative impact on quality of life. For simplicity, PCS will be the term used throughout this article. Common symptoms of PCS include fatigue, post-exertional malaise, (also termed post-exertional symptom exacerbation), dyspnea, anxiety, depression, cognitive impairment or “brain fog”, and in some instances, autonomic dysfunction, including postural orthostatic tachycardia syndrome.^3^ The World Health Organization (WHO) defines PCS as “the continuation or development of new symptoms 3 months after the initial SARS-CoV-2 infection, with these symptoms lasting for at least 2 months with no other explanation.”^4^ A recent systematic review and meta-analysis (*n* = 735,006) shows that 45% of individuals who survive a COVID-19 infection will have at least 1 unresolved symptom at 126 days (~4 months) post-COVID infection regardless of whether they required hospitalization.^5^

There is a substantial body of evidence showing pulmonary rehabilitation improves exercise tolerance, dyspnea, anxiety, depression, and health-related quality of life (HRQoL) for respiratory conditions^6^ including chronic obstructive pulmonary disease,^7^ bronchiectasis,^8^ interstitial lung disease,^9^ chronic asthma,^10^ and pulmonary hypertension.^11^ Less is known about the effectiveness of PR in people with PCS. The timely advent of telehealth has equipped clinicians with the technology and skills necessary to provide telerehabilitation for populations with chronic respiratory conditions and has been shown to be safe and effective.^12^ However, there is limited robust evidence on the effects of exercise rehabilitation (either delivered face-to-face or via telerehabilitation) for people who have respiratory specific sequalae of PCS, which commonly includes dyspnea, chest pain, and cough^13^ and, in some cases, a dysfunctional pattern of breathing.^14^

Since WHO has urged the prioritization of PCS rehabilitation for those experiencing medium and long-term consequences of COVID infection,^15^ some limited, yet encouraging, evidence has emerged. Multiple reviews have shown that PR reduces post-COVID sequelae such as dyspnea^16–18^; improves HRQoL^18^; increases exercise capacity^16^; and promotes the feasibility of service delivery through existing PR pathways.^16^^,^^19^ However, inclusion of primarily low quality studies is a limitation reported by most reviews. To the best of the authors’ knowledge, there are currently no published randomized controlled trials (RCTs) of supervised pulmonary telerehabilitation for people with respiratory specific symptoms of PCS. The aim of this study is to evaluate the effect of a 4-week, twice weekly supervised pulmonary telerehabilitation program on exercise capacity, symptoms, cognition, anxiety, depression, HRQoL, and fatigue compared with usual medical care for people with predominantly respiratory sequelae of PCS.

## Methods

### Participants

Participants who had a COVID-19 infection, confirmed via a rapid antigen test or polymerase chain reaction test, and who were either hospitalized or managed in the community, and who meet the following criteria will be invited to participate in the study.

Inclusion criteria will be people with respiratory sequelae of PCS attending a post-COVID Respiratory Clinic at 2 metropolitan tertiary hospitals in Sydney, Australia; aged 18 years or above; identified by their treating physician as suitable for rehabilitation; and able to provide informed consent. Exclusion criteria will be people with a severe COVID-19 infection admitted to intensive care unit and who develop post–intensive care unit syndrome (such people usually require rehabilitation and therefore cannot take part in a trial where they may be randomized to a control group); acute symptoms of any illness where exercise is not recommended; medically unstable as diagnosed by their treating physician; pregnant or post-partum women; no access to appropriate technology (eg, internet, computer, or tablet); difficulty understanding English and unable to access an interpreter; or severe cognitive impairment or other comorbidities which would make remote exercise unsafe as assessed by the referring physician.

People who meet the inclusion criteria based on their clinic visit will be invited to participate. If agreeable, they will be provided with a participant information sheet and will be contacted by a member of the research team to answer any questions and to complete an online consent form.

### Study Design

This will be a prospective, multi-site, RCT with assessor blinding, and will follow the Guidelines for Reporting Outcomes in Trial Reports (CONSORT). Participants will be randomized into the intervention group (IG) or the control group using a concealed computer-generated sequence, through a secure data management software, Research Electronic Data Capture (REDCap), with a minimization algorithm, stratifying for age (≤50 or >50 years) and sex (male/female). The study has been approved by the Sydney Local Health District (SLHD) Human Research and Ethics Committee and registered on the Australian and New Zealand Clinical Trials Registry (ACTRN 12622000355774).

### Intervention Group

The IG will receive telerehabilitation via videoconferencing (Zoom Video Communications Inc., San Jose, CA, USA) twice per week for 4 weeks, supervised by a physical therapist experienced in remote exercise rehabilitation and symptom monitoring. The program will consist of aerobic exercise (including marching on the spot and multi-directional stepping exercises with or without added arm movements, similar to a gym-based aerobics class) with intensity based on the participant’s level of breathlessness or fatigue, whichever is highest, aiming for 2 to 4 (“slight” to “somewhat severe”) on the 0 to 10 category ratio scale.^20^ A modified category-ratio scale will be used for assessing fatigue (Suppl. Material 1). Additionally, we will ask participants to rate their worst fatigue in the period 12 to 24 hours post-exercise using this scale. In situations where fatigue increases by >2 points from a participant's pre-exercise fatigue rating, intensity prescribed during the following session will be reduced to avoid future post-exertional malaise. Strength training will consist of exercises using body weight (eg, squats, sit-to-stand), hand weights using cans of food, bags of rice, etc. (eg, biceps curls, shoulder flexion/abduction, upright row), or resistance bands. Strength exercises will be performed in 1-minute intervals and will be based on the individual’s fatigue and dyspnoea. The exercise program will last for 40 minutes, approximately 25 minutes for aerobic training and 15 minutes for strength training and will be in groups no greater than 5 participants, who will always be visible on the computer screen. Participants will be able to see and converse with the physical therapist and other participants during the sessions in real-time through the videoconferencing technology. The specific resistance and aerobic exercises to be prescribed with possible progressions and regressions for each are presented in Supplemental Material 2.

#### Fidelity

To ensure that the intervention is delivered consistent with the protocol in this multi-site (4 sites) study, the chief investigator will regularly log into a telerehabilitation session at each site. Any deviations from the study protocol will be discussed immediately after the session so that the intervention is delivered uniformly at all sites.

#### Safety Considerations

At the initial assessment prior to the first exercise session, the physical therapist will do a safety check by visually scanning the participant’s environment. At the commencement of an exercise session, if concerning symptoms are evident, the participant will not undertake the session and will be asked to present to their general practitioner or the emergency department, depending on the severity of symptoms. During a session, if the participant experiences a change in symptoms or has severe breathlessness or fatigue, they will be advised to stop exercising. All participants will be asked to report any new symptoms or change in symptoms before they start an exercise session. If fatigue was worse after the previous session, the exercise intensity will be reduced by using more rests, lighter weights, slower pace, and/or modified exercises. Participants will be asked to have a working phone nearby and the physical therapist will have the participant’s phone number available in case the internet drops out. In the case of a serious adverse event the physical therapist will call emergency services immediately.

Education will be provided by the physical therapist at rehabilitation sessions. Topics will include strategies to manage fatigue; pacing with daily activities; staying physically active; managing anxiety and depression; and returning to work. The education will be based on the WHO Pamphlet *Support for Rehabilitation: Self-Management after COVID-19-Related Illness*^21^ (Suppl. Material 3) and participants will be referred to the Lung Foundation Australia (LFA) *Understanding Long COVID* booklet^22^ (Suppl. Material 4). At the end of the 4-weeks, participants in the IG will receive advice on continuing with physical activity. All participants will be invited to complete a survey to help the investigators gain insight into the participants’ experiences with telerehabilitation.

#### Participant Interviews

At the end of the intervention period, between 15 and 20 participants in the IG will be invited to take part in a semi-structured, 30-minute interview via videoconferencing technology to gain a deeper understanding of their experiences and opinions about telerehabilitation. Participants will be invited to undertake an interview irrespective of whether they completed 8 sessions of the intervention. The interviewer will be a clinician independent of the treating and research team and will ask questions about barriers and enablers to participation in telerehabilitation for people with PCS.

### Control Group

The control group will receive usual medical care as well as a copy of the WHO Pamphlet.^21^ At the end of the 4-week control period, participants in the control group will be invited to cross-over into the IG. The cross-over is to encourage recruitment since the advice from those with lived experience of PCS was that participants needed the option to receive telerehabilitation after the control period. Additionally, as research in telerehabilitation for PCS is currently very limited, the crossover will provide increased data on rehabilitation outcomes.

### Outcome Measures

**Figure 1:**
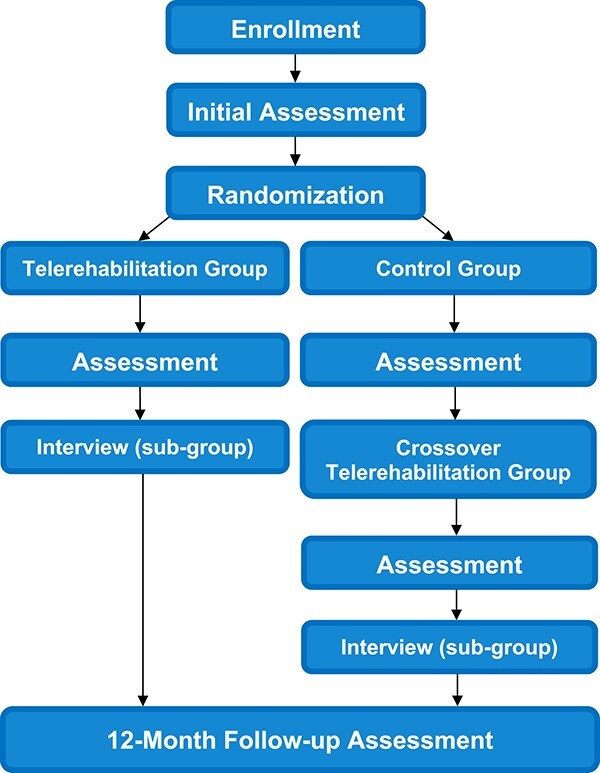
Study flow chart.

**Figure 2:**
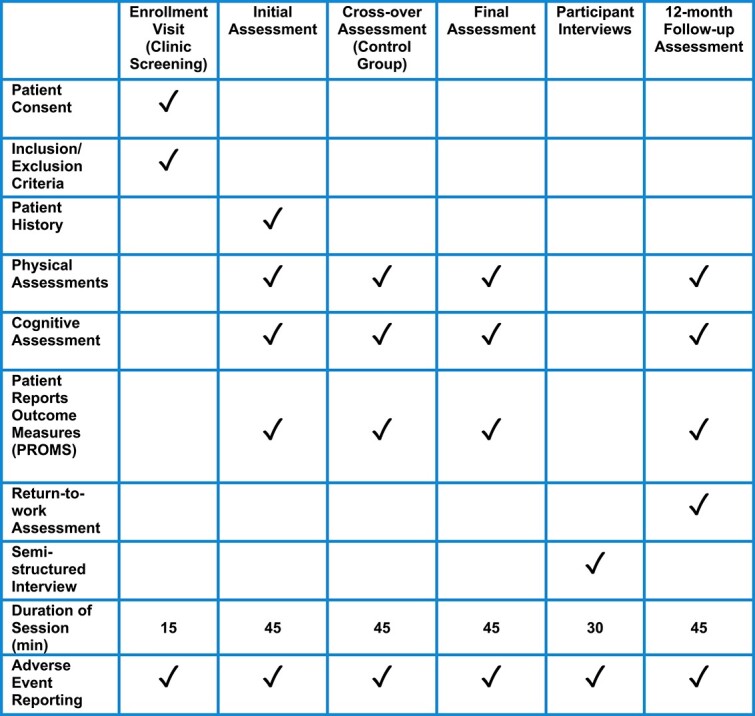
Study visits timeline.

Following recruitment, baseline outcomes will be collected at an initial videoconference assessment. At all follow-up assessment times (Figs. 1 and 2), the physical outcomes will be collected by a physical therapist who is blind to group allocation. Participants will be asked not to divulge their group allocation. Outcome measures are outlined below.

#### Physical Outcomes

Exercise capacity will be measured using the 1-minute sit-to-stand test (1-minSTST)^23^ (primary outcome measure). The 1-minSTST is considered a measure of aerobic capacity and has a moderate correlation with 6-minute walk test distance.^24^ The 1-minSTST has been validated and is widely used across the age-span.^25^ Functional lower limb strength will be measured by the 5 repetition sit-to-stand test. Recent evidence demonstrates that the use of sit-to-stand tests using telehealth technology is safe, reliable, and valid.^26–29^

During screening, all participants will have previously completed a 1-minSTST with pulse oximetry monitoring, face-to-face under the supervision of a physical therapist in a post-COVID Respiratory Clinic to check for safety. During the study the sit-to-stand tests will be supervised via videoconferencing by an experienced physical therapist and will allow comparisons to be made under the same conditions at all assessment timepoints. While a 46 cm-height chair without arm rests is standard for these tests,^23^ a chair closest to this height (measured by the participant) will be used and the same chair will be used at each assessment.

#### Cognitive Function

Cognitive function will be measured using the Montreal Cognitive Assessment suitable for remote assessment (MoCA-BLIND; MoCA Test Inc., Québec, Canada)^30^ and will be administered by a physical therapist. Three alternative MoCA-BLIND versions (Version 7.1, 8.2, and 8.3) will be used for repeat assessments to reduce a possible practice effect.

#### Patient Reported Outcomes

The following questionnaires will be emailed to the participant through REDCap and will be completed at each assessment timepoint (Fig. 2). Participants in both groups will be asked to complete a weekly symptom diary via REDCap.

HRQoL will be measured using the 36-Item Short-Form Health Survey (SF-36)^31^ which comprises 36 questions across 8 domains of health. Anxiety and depression will be measured using the Hospital Anxiety and Depression Scale^32^ and the Kessler Psychological Distress Scale (K6+).^33^ The Hospital Anxiety and Depression Scale is a 14-item validated questionnaire^34^ and the K6+ is a reliable and valid 6-item questionnaire.^35^ Fatigue will be measured using the 9-item Fatigue Severity Scale^36^ which measures both severity of an individual’s fatigue and the effect of fatigue on their activities of daily living. Respiratory symptoms will be measured using the Chronic Obstructive Pulmonary Disease Assessment Test.^37^ Although designed for people with chronic obstructive pulmonary disease, the Chronic Obstructive Pulmonary Disease Assessment Test is widely used for many respiratory conditions as a measure of the presence and severity of respiratory symptoms such as breathlessness, cough, chest tightness, and sputum production.

Composite outcomes will be measured using the COVID-19 Yorkshire Rehabilitation Scale (YRS-19)^38^ which is a comprehensive COVID-specific patient reported measure that provides a score for several outcomes related to the domains of “symptoms severity” and “functional disability”.

At the 12-month follow-up assessment, participants will be asked to complete all previous assessments as well as the return-to-work questions from the International Severe Acute Respiratory and emerging Infection Consortium (ISARIC) questionnaire.^39^ The 12-month follow-up is exploratory and will evaluate any ongoing effects of the intervention, as well as natural recovery in participants in the control group who did not cross-over.

### Sample Size

A total of 42 participants will be required to have an 80% chance of detecting, as significant at the 5% level, a between-group difference of 3.5 repetitions in the 1-minSTST (primary outcome), which is the minimal clinically important difference, using a standard deviation of 4 repetitions.^23^ To account for a 15% dropout, 48 participants will be recruited.

The sample size for the semi-structured interviews will be approximately 15 to 20 participants. Interviews will continue until thematic saturation.

### Statistical Analysis

Statistical analysis will be performed using IBM SPSS version 28 (IBM Corporation, Armonk, NY, USA). Intention-to-treat analysis of between-group changes at the end of the initial intervention/control period will use linear mixed effects model analysis of variance for the primary and secondary outcome measures, with adjustments for any statistically significant differences in baseline covariates between the groups. Maximum likelihood estimation through the linear mixed effects model analysis of variance will be used for missing data. Following an intention-to-treat analysis, a “per protocol” analysis will be undertaken using the same statistical methods. Post-hoc analyses will also be undertaken to establish characteristics of responders to the intervention. Analysis of the data from the cross-over group will use repeated measures analysis of variance to evaluate the within-group change due to the intervention. Data analysis at the 12-month follow-up will be exploratory. If adequate numbers in the control group do not cross-over to the intervention, a between-groups analysis will be undertaken. However, it is anticipated that the majority of participants in the control group will cross-over into the IG, in which case the analysis will evaluate the change from completion of the intervention to the 12-month timepoint. The level of significance for all outcomes will be set at an alpha of <0.05.

The semi-structured interviews will be transcribed and coded using NVivo 9 (QSR International, Melbourne, Vic., Australia). Thematic and descriptive qualitative analysis^40^ will be used to identify key themes regarding participant opinions, and perceived barriers and facilitators to participation in the telerehabilitation program. At least 3 investigators will review the coding to ensure it is insightful. Any discrepancies will be discussed by the research team and the coding revised accordingly.

### Data Management

Electronic data will be securely stored on a password-protected data management software, REDCap. Study outcome data will be separated from any identifiable information. Hard copies of original data will be stored in a locked filing cabinet in a locked office at the site of participant recruitment. A data safety and monitoring board will be established to review and evaluate the accumulated study data for participant safety, study conduct, and progress. The data safety and monitoring board will consist of 2 researchers and a biostatistician, all of whom will be independent of the study and sponsor. The board will meet twice annually.

### Role of the Funding Source

The funder will have no role in the design, conduct, or reporting of this study.

## Dissemination

The authors intend to publish the findings in peer-reviewed journals as well as present data at conferences.

## Discussion

Given the recency of COVID-19 as a global pandemic, evidence for the most appropriate rehabilitation for individuals reporting PCS is in evolution. Guidelines on rehabilitation have mainly focused on consensus statements and expert opinion and are now adapting to incorporate a stronger evidence-base. Several Australian and international guidance documents on COVID-19 rehabilitation and management have common rehabilitation themes. Documents for guiding Australian practice have been developed by the National COVID-19 Clinical Evidence Taskforce,^41^ the NSW Agency for Clinical Innovation (ACI),^42^^,^^43^ and Exercise and Sport Science Australia.^44^ Guidance documents from International bodies include the WHO,^21^^,^^45^ the National Institute for health and Care Excellence (NICE),^46^ World Physiotherapy,^47^ the Chartered Society of Physiotherapy,^48^ the European Respiratory Society (ERS),^49^ and others.^50–53^ Themes include validating patient experiences; ensuring rehabilitation is personalized and needs based; providing equitable and accessible care; fostering a multidisciplinary approach; and assessing symptoms or conditions that could make rehabilitation unsafe (such as postural orthostatic tachycardia syndrome or post-exertional symptom exacerbation) or requiring the need to proceed with appropriate modification.

Several prospective cohort studies have demonstrated the effectiveness of face-to-face outpatient rehabilitation on PCS symptoms, exercise capacity, and HRQoL,^54–57^ although, since these studies were not RCTs, the effect of natural recovery is unknown. A number of RCTs have evaluated exercise interventions. One study evaluated an unsupervised 6-week, home-exercise program delivered via smartphone and demonstrated improvements in strength and HRQoL^58^; 2 separate inspiratory muscle training trials of 8 and 12 weeks, respectively, demonstrated improvement in breathlessness, exercise capacity, and HRQoL compared with usual care for people reporting post-COVID dyspnea^59^^,^^60^; and a trial evaluating very short duration (10 minutes) “respiratory rehabilitation” involving the use of a threshold positive expiratory pressure device, forced repeated coughing exercise (3 sets of 10 coughs), diaphragmatic breathing, and upper limb dynamic stretching, demonstrated improvements in respiratory function, HRQoL, and anxiety.^61^ The rationale for this study is that there are no published RCTs that have evaluated telerehabilitation including exercise training (similar to that which is provided in a PR program) and education for people with PCS with specific respiratory sequelae. The importance of the study is that, due to the high prevalence of people with PCS, robust research is needed to investigate rehabilitation programs that address the debilitating symptoms of PCS.

The optimal length of rehabilitation programs for people with PCS is unknown. We have chosen to evaluate a short-duration (4-week) program. A number of studies of exercise training provide evidence of improvements in exercise capacity and quality of life in short-duration programs.^62^^,^^63^ For example, in people with PCS, intensive PR of 3 weeks was effective in improving exercise capacity and quality of life (although this was a 5 day per week intervention)^57^ and a 4-week unsupervised program with video-assisted, individualized exercises demonstrated improvements in functional capacity.^63^ Additionally, in people with chronic obstructive pulmonary disease, the greatest statistically significant improvements in exercise capacity occurred within the first 4 weeks of a PR program.^62^ The short duration may also be more acceptable to those with PCS who remain in the workforce with limited time for rehabilitation.

There is growing evidence for the effectiveness of telerehabilitation, which will be used in our RCT. A Cochrane review has shown that telerehabilitation programs achieve comparable results with traditional in-person outpatient PR for people with chronic lung disease, and may even have a higher completion rate.^12^ A case series of patients with PCS has demonstrated that rehabilitation via videoconferencing was feasible and safe.^64^ The proposed telerehabilitation program, if effective, would make rehabilitation more widely accessible for those with PCS for whom access to face-to-face rehabilitation may be geographically challenging. Such programs could provide a mode of rehabilitation less affected by possible future mobility restrictions and pandemic-associated lockdowns.

This multi-site RCT will provide important evidence to determine appropriate rehabilitation interventions for the large number of individuals with PCS, particularly those with respiratory sequelae.

## Supplementary Material

2023-0677_R2_Supplementary_Material_1_pzae080

2023-0677_R2_Supplementary_Material_2_pzae080

2023-0677_R2_Supplementary_Material_3_pzae080

2023-0677_R2_Supplementary_Material_4_pzae080

## Data Availability

Data supporting the findings of the study will be published in a peer-reviewed journal. The data files may be available from the authors upon reasonable request, subject to permission being granted by the Sydney Local Health District (SLHD) Human Research and Ethics Committee.

## Clinical Trial Registration

The study is registered on the Australian and New Zealand Clinical Trials Registry (ACTRN 12622000355774).
