# Supplementary material for: Effect of a 4-Week Telerehabilitation Program for People With Post-COVID Syndrome on Physical Function and Symptoms: Protocol for a Randomized Controlled Trial
Source: Phys Ther. 2024 Jun 29;104(9):pzae080. doi: 10.1093/ptj/pzae080 (PMC11443032; doi:10.1093/ptj/pzae080)
Supplement: 2023-0677_R2_Supplementary_Material_1_pzae080 [file 2023-0677_r2_supplementary_material_1_pzae080.pdf]

Supplementary Material 1: Scale for assessing fatigue

Fatigue Scores	
0	No Fatigue at all (Energetic)
0.5	Very, very slight fatigue (just noticeable)
1	Very slight fatigue
2	Slight Fatigue
3	Moderate fatigue
4	Somewhat severe fatigue
5	Severe fatigue
6	
7	Very severe fatigue
8	
9	Very, very severe fatigue (almost maximal)
10	Maximal fatigue
