# Supplementary material for: Effect of a 4-Week Telerehabilitation Program for People With Post-COVID Syndrome on Physical Function and Symptoms: Protocol for a Randomized Controlled Trial
Source: Phys Ther. 2024 Jun 29;104(9):pzae080. doi: 10.1093/ptj/pzae080 (PMC11443032; doi:10.1093/ptj/pzae080)
Supplement: 2023-0677_R2_Supplementary_Material_2_pzae080 [file 2023-0677_r2_supplementary_material_2_pzae080.pdf]

Supplementary Material 2: Resistance and aerobic exercises with potential progressions and regressions

Aerobic Training		
**Progression**	**← Exercise →**	**Regression**
Jogging on the spot OR holding hand weights	Marching on the spot	Marching while holding a chair OR marching in a seated position
Increased pace OR increased knee height OR holding hand weights	Alternating High Knee flexion on the spot	Decreased pace OR holding a chair OR marching in a seated position
Larger side-stepping and arm abduction OR faster pace OR while holding hand weights	Star steps (side-stepping while synchronously abducting arms)	Smaller side-stepping and arm abduction OR slower pace OR side stepping without arm movements
Larger steps OR adding synchronised arm abduction movements OR faster pace OR holding hand weights	Square stepping (stepping in a square pattern)	Smaller steps OR slower pace OR replaced with marching (to remove cognitive component)
Larger steps OR faster pace OR while holding hand weights	Forward and backward stepping (alternating leading foot and number of steps)	Smaller steps OR slower pace OR replaced with marching (to remove cognitive component)
**Resistance Training**		
**Progression**	**← Exercise →**	**Regression**
Bilateral rather than alternating unilateral OR heavier weight	Standing Bicep Curls – Alternating unilateral with HW	Seated OR lighter weight/no weight
Holding a weight OR from a lower chair	Sit-to-stands (BW)	Increase chair height
Single leg OR on a step or a book	Standing Calf Raises (BW)	Holding onto a chair for balance OR seated with a weight placed on the knees
A full squat (i.e., larger hip/knee range of motion) OR holding a weight to the chest	Mini-Squats (BW)	Holding onto a chair for support
With an increased range of motion (i.e., lifting the moving leg higher)	Single-leg Standing hip abduction (BW)	With a decreased range of motion OR holding onto a chair
With a heavier weight	Standing Shoulder Press – Bilateral (HW)	With lighter weight/no weight OR performed seated
With an increased range of motion (i.e., lifting the moving leg higher)	Single-leg Standing hip extension (BW)	With a decreased range of motion OR holding onto a chair
With a synchronised step forward and back OR heavier weight	Standing Forward Punches (HW)	With a decreased weight/no weight OR seated
With a heavier weight	Single-arm Bent-over Rows (holding chair) (HW)	With a lighter weight/no weight
With a heavier weight	Standing Shoulder Raises (flexion and abduction to 90 degrees) (HW)	With a lighter weight OR whilst seated OR with <90 degrees range of motion
With a heavier weight	Upright Row (HW)	With a lighter weight OR whilst seated
