# Supplementary material for: Effect of a 4-Week Telerehabilitation Program for People With Post-COVID Syndrome on Physical Function and Symptoms: Protocol for a Randomized Controlled Trial
Source: Phys Ther. 2024 Jun 29;104(9):pzae080. doi: 10.1093/ptj/pzae080 (PMC11443032; doi:10.1093/ptj/pzae080)
Supplement: 2023-0677_R2_Supplementary_Material_4_pzae080 [file 2023-0677_r2_supplementary_material_4_pzae080.pdf]

Understanding **long COVID**

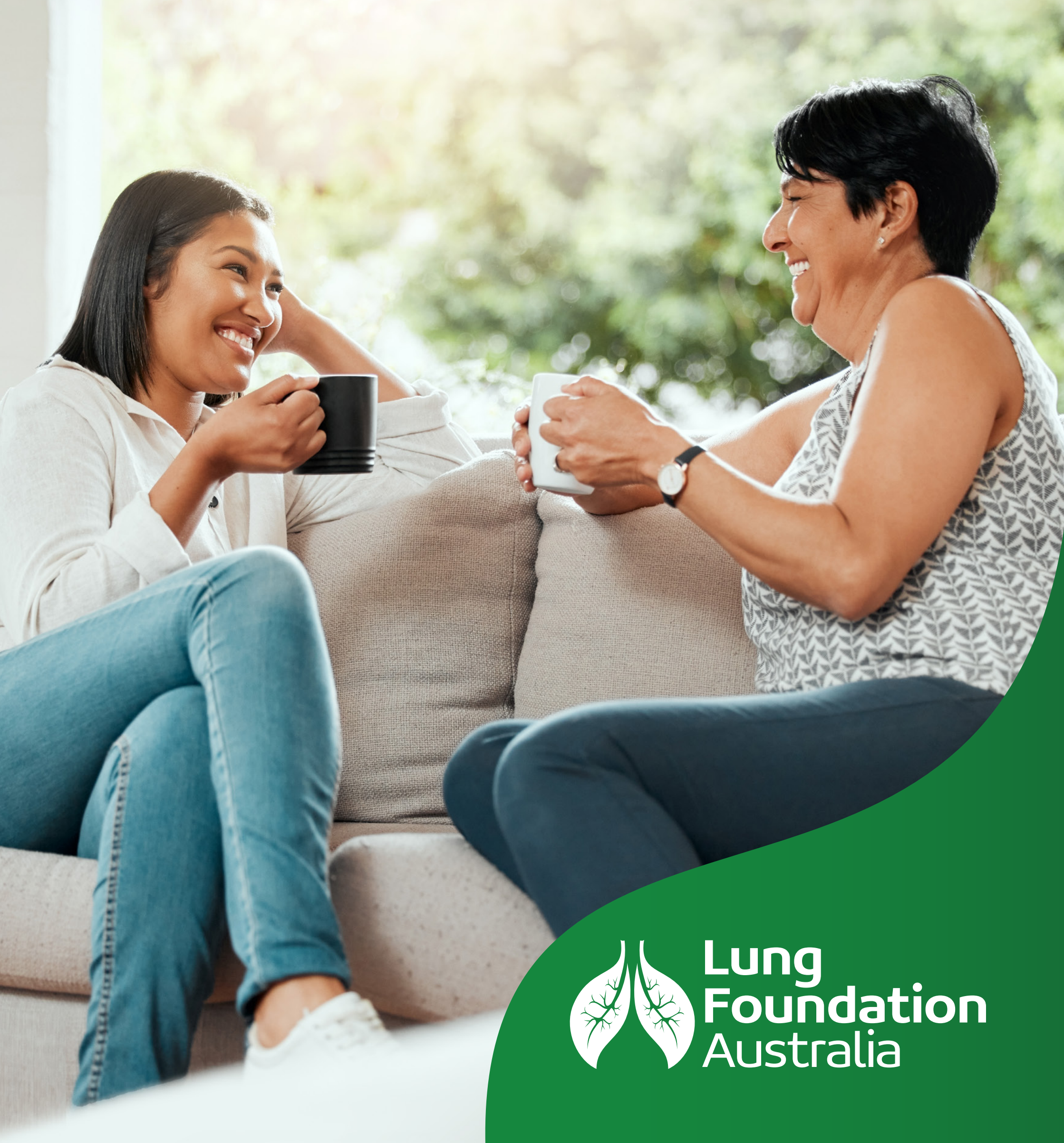

Lung Foundation Australia

Title: Understanding long COVID

For people living with long COVID, their families, carers and friends.

First published: May 2023

© Lung Foundation Australia

ISBN: 978-0-6480834-7-4

This booklet was developed with the input of the following healthcare professionals and consumers with a special interest in long COVID. We thank all contributors for their time and efforts in creating this resource.

- Prof Jennifer Alison, Respiratory Physiotherapist
- Ellie (Elham) Asgary, Long Covid Clinical Nurse Consultant
- Karen Blazquez, Speech Pathologist
- A/Prof Philip N Britton, Paediatrics and Infectious Diseases
- A/Prof Lucy Burr, Respiratory Physician
- A/Prof Anthony Byrne, Thoracic Physician
- Vincent Cram, Clinical Psychologist
- Dr Susan Graham, Rehabilitation Physician
- Lisa Hankey, Consumer Representative
- Judy Little, Consumer Representative
- Jennifer Marty, Consumer Representative
- Dr Fiona Raciti, General Practitioner
- A/Prof Debbie Rigby, Advanced Practice Pharmacist
- Mary Roberts, Clinical Nurse Consultant
- Bill Simpson-Young, Consumer Representative
- Michelle Thompson, Consumer Representative
- Michael Wai, Clinical Psychologist
- Prof Peter Wark, Respiratory and Sleep Specialist
- Elizabeth Wells, Consumer Representative
- Scott Willis, Physiotherapist

Note to reader:
This information is a general guide only and is not intended to be a substitute for professional medical advice or treatment. While all care is taken to ensure accuracy at the time of publication, Lung Foundation Australia and its members exclude all liability for any injury, loss or damage incurred by the use of or reliance on the information provided. Always consult your healthcare professional on matters that affect your health.

Lung Foundation Australia have more information about COVID-19 and long COVID online: **lungfoundation.com.au**

Lung Foundation Australia

Contents

Introduction 4
What is long COVID? 6
Diagnosing long COVID 7
Getting help for long COVID 8
Tips for healthcare appointments 10
Talking to family and friends 11
Symptoms and management tips 12
- Fatigue 14
- Post-Exertional Symptom Exacerbation (PESE) 15
- Breathlessness 19
- Cough 21
- Brain Fog 23
- Cardiac (heart) symptoms 24
- Postural Orthostatic Tachycardia Syndrome (POTS) 25
- Sleep 26
- Emotional wellbeing 27
- Pain 29
- Gastrointestinal symptoms 29

Other tips 30
- Returning to exercise 30
- Maintaining a healthy diet 30
- Stopping smoking and vaping 30
- Reducing alcohol consumption 30
- Returning to work 31
- Returning to school 31

Introduction

This booklet contains information for people who have been diagnosed with, or suspect they may have, post Acute COVID-19 or chronic COVID condition hereafter called, “long COVID”. It has been designed to help you navigate your long COVID journey and outlines common symptoms, provides helpful self-management tips, treatment information and expert advice on how to discuss your condition with healthcare professionals, family and friends.

The information has been developed by a group of healthcare professionals with a special interest in long COVID as well as people living with long COVID and other related conditions. It is a general guide and does not replace the information provided by your healthcare team.

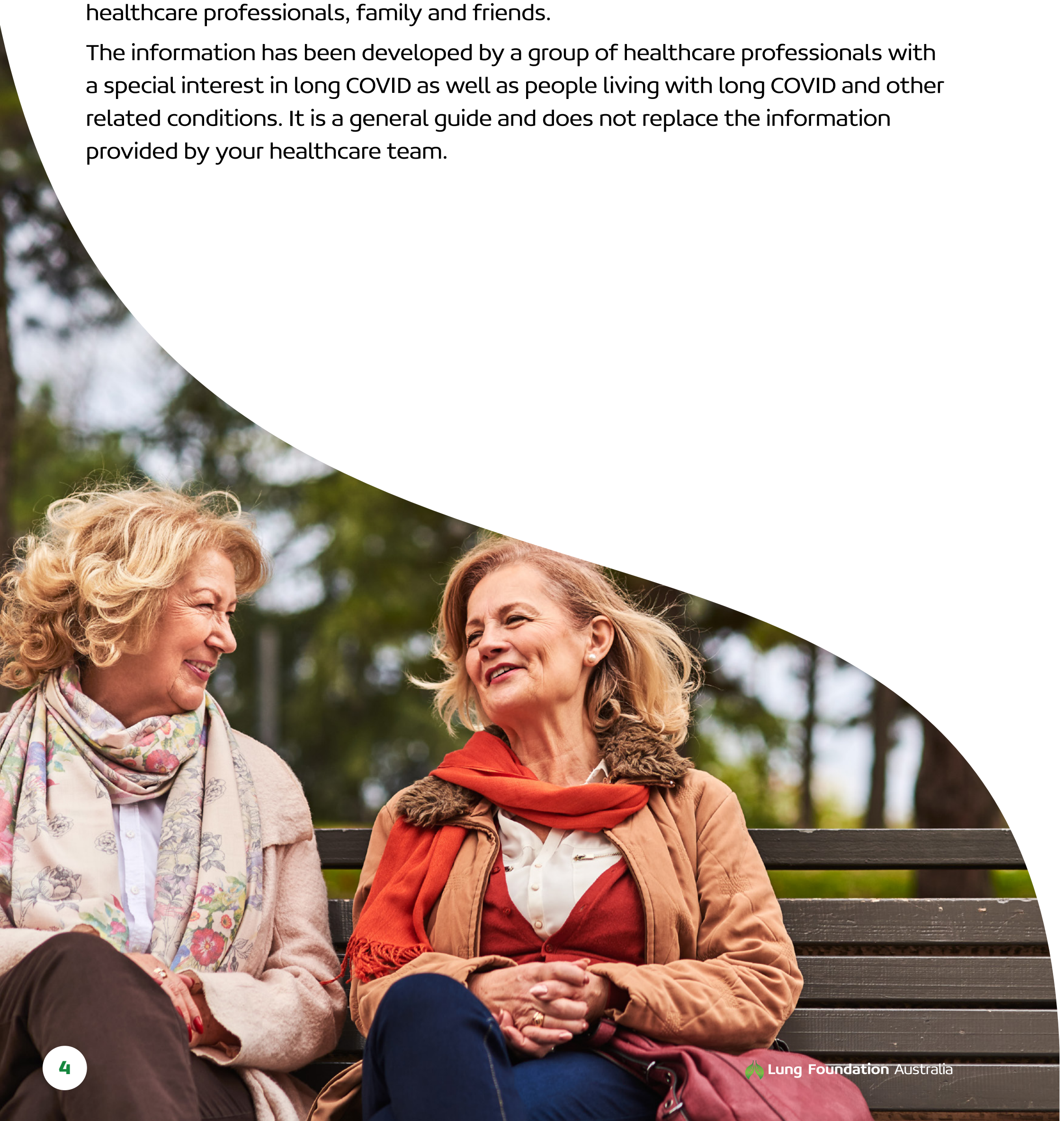

COVID-19 timeline

One month

Acute COVID-19

When you first test positive to COVID-19 and have initial symptoms. Most people will recover from acute COVID-19 within a week or two.

Two months

Prolonged COVID-19

Some people will have the acute symptoms of COVID-19 for more than 2-4 weeks. Some may continue to test positive on RAT or PCR testing. Prolonged COVID-19 is not a post-viral illness like long COVID. You are still experiencing symptoms as you have not yet recovered from the initial infection.

Three months +

Long COVID

Experiencing symptoms three months after infection with COVID-19 is a commonly used criterion for diagnosis, provided these symptoms have no other underlying cause as an explanation.

This timeline includes definitions of acute COVID-19, prolonged COVID-19 and long COVID. Keep in mind that these are approximate, and you may experience long COVID symptoms before getting a formal diagnosis. In general, those people with a greater number of, or more severe symptoms at the time of their acute COVID-19 illness will be more likely to progress to long COVID.

What is long COVID?

Long COVID is a chronic health condition that is caused by infection with the SARS-CoV-2 virus and lasts at least three months in duration. It is a new and complex illness that patients and healthcare professionals are beginning to better understand. Long COVID can impact many organs of the body and affects everyone differently. For some people it may cause symptoms such as breathlessness, cough, hoarse voice and fatigue whereas other people may experience memory or thinking problems (often referred to as “brain fog”).

Regardless of specific symptoms, long COVID often affects a person’s ability to perform their usual activities day-to-day and live their life as they normally would. Symptoms may be mild, moderate, or so severe that the person is unable to work or study.

Using this definition, current prevalence estimates of long COVID in Australia range from 5 – 10% of COVID-19 cases. The risk of long COVID is likely to be lower for people who have been vaccinated or had later variants of COVID-19 – for example Omicron.

Everyone’s experience of long COVID is different. The World Health Organization has recorded over 200 different long COVID symptoms. Everyone’s individual circumstances will vary. The best way to manage long COVID is to work with a healthcare professional to develop a tailored management plan to help your recovery.

Diagnosing long COVID

There is currently no single test to diagnose long COVID. Confirming whether your symptoms are due to long COVID will involve a series of questions and tests that help your healthcare professional understand how your body has changed since having COVID-19 and what management strategies are likely to be of benefit. The questions and tests will vary depending on what is deemed necessary depending on your symptoms, but may include a series of questions, such as:

- **When did you have COVID-19?**
- **Do you have any pre-existing medical conditions?**
- **What symptoms do you have?**
- **What changes have you noticed since having COVID-19?**
- **How have your symptoms impacted your work and lifestyle?**
- **What are your goals for treatment?**
- **How have you been managing your symptoms to date – what has worked and what hasn’t worked?**

Along with tests to investigate your symptoms and rule out other possible causes. These may include:

- **Blood tests**
- **Blood pressure and heart rate tests**
- **Lung function tests**
- **X-rays**
- **Electrocardiography.**

Collectively, the results of these questions and tests can help your healthcare professional to better understand your symptoms and help create a tailored management plan.

Before seeing your healthcare professional, it may be useful to think through your top three or five symptoms that are most affecting your life so these can be prioritised.

Getting help for long COVID

If you are experiencing long COVID symptoms it is important to connect with healthcare professionals to get help in managing your condition. Usually your **General Practitioner (GP)** will be your initial point of contact. They will be able to help you create a tailored plan for monitoring and managing any ongoing symptoms. As required, your GP will be able to prescribe medications and act as a referral point to other medical specialists and support. This may include organising Mental Health Treatment Plans and Chronic Disease Management plans to help you access Medicare-subsidised allied health services where eligible.

You may engage with the following healthcare professionals:

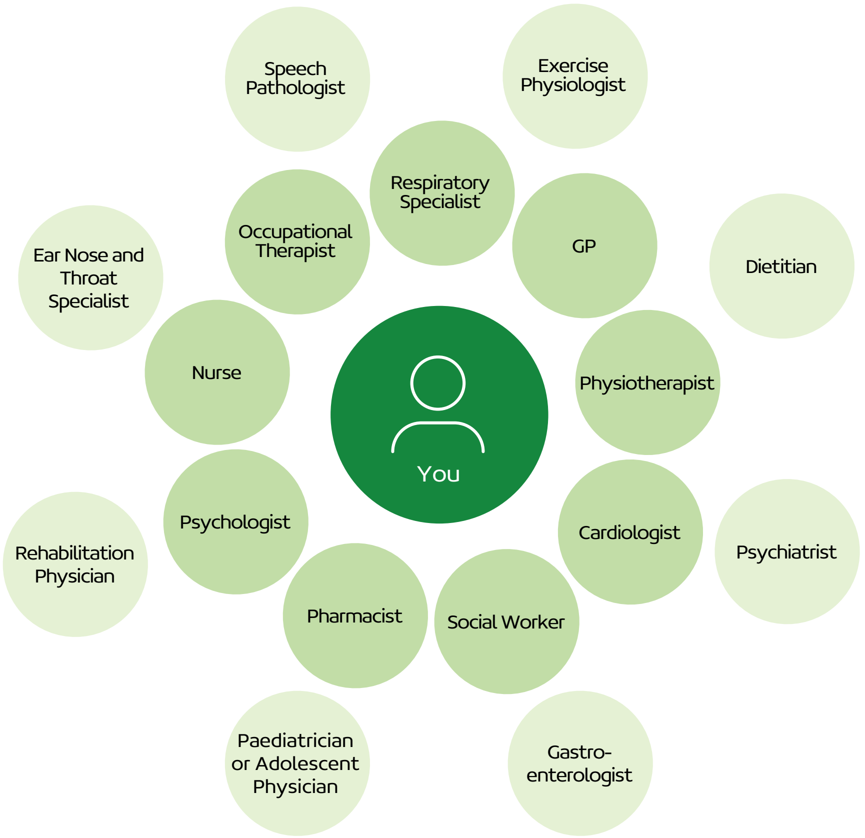

Your GP can discuss the role of each of these health professionals in your care.

Some states and territories have set up dedicated clinics to support people to manage long COVID. To see whether there are long COVID clinics in your area, view our state and territory information section on **Lung Foundation Australia's COVID-19 web page**.

Getting a second opinion

It may be important to speak to more than one healthcare professional regarding your diagnosis and recommended management plan.

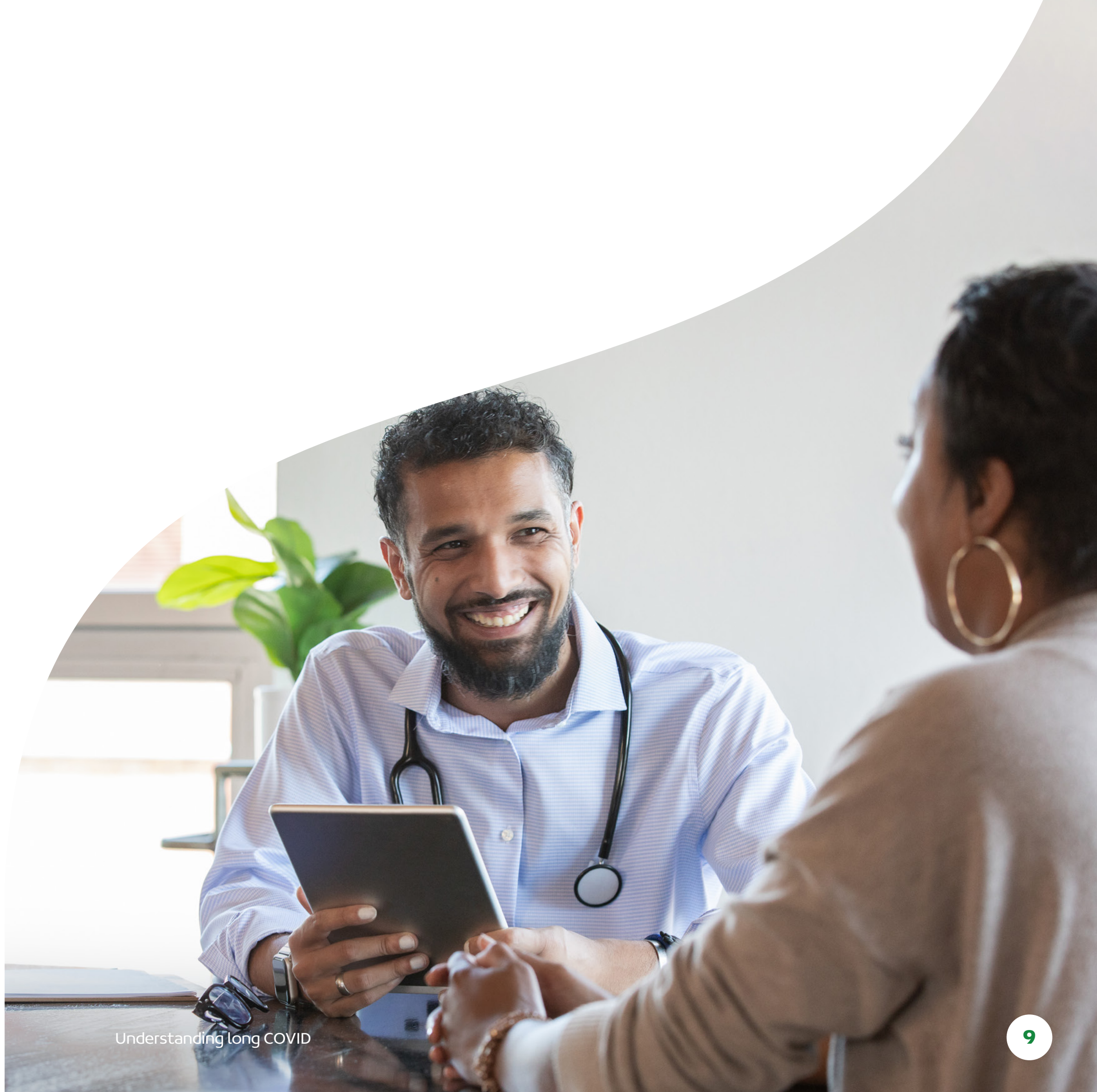

Tips for healthcare appointments

Ongoing COVID-19 symptoms such as brain fog can be exhausting and may make visiting a healthcare professional a difficult experience. But there are things you can do to ease the process.

Some tips include:

- **Write down your symptoms and examples of how they affect your daily life** (e.g., symptom: I feel weak and tired; examples: I can't walk up a flight of stairs without stopping and resting, my friends have commented on it) to help you remember everything you'd like to discuss.
- **Keep a symptom diary or write any changes down as they happen** to help you monitor your symptoms over time.
- Consider **bringing someone with you** to your appointment, such as a friend or family member, for support. They can also listen and take notes for you.
- If you don't understand something, **ask your healthcare professional to repeat the information** or explain it in a different way. Do not avoid asking questions because you're afraid or embarrassed. Your healthcare team is there to help you.
- Ask your healthcare professional to help you by **writing things down during your appointment**. That way you will have a resource to look back at and don't need to rely on remembering everything that is said.
- Consider **booking a long appointment** to ensure you will have enough time to go over everything on your list.

> *"When you have long COVID you can't exactly avoid the crashes, the thing that does change is how severe they are and the quality of life you get in between. Pacing gives me a quality of life that I didn't have before and allows me to do things I enjoy – like gardening."*
>
> **Lisa, TAS resident living with long COVID.**

Talking to family and friends

Long COVID symptoms can be quite confusing, and the impacts may be hard for your family and friends to understand. It may be exhausting initially, but it is important to take time to communicate with your loved ones about how you feel and explain how your symptoms are affecting you. Have a think about how you want to be supported and explain this clearly. The support you need is individual and may range from help with tasks of daily living, to just having a supportive listener. There is no right or wrong way to go about this, but to be able to get the help you need you will have to communicate effectively. Consider sharing long COVID resources, such as this one, with your loved one so they can better understand your condition.

It may also be beneficial for you to link in with a peer support network. Contact Lung Foundation Australia on freecall **1800 654 301** to find out more.

Along with asking for help from family or friends, it can be good to consider whether there are any services you could use to assist with getting your essential activities completed each week. A good example of this is a food or grocery delivery service.

> *"I was so exhausted that I couldn't even imagine doing actual exercise - it felt like I was struggling to drag my body around the house for just basic things. Then I started with just six to eight minutes every few days, doing a seated Zumba routine, after consulting with the Community Rehabilitation Physiotherapist, and being very careful to stop well before I got tired. It was actually a fun challenge, and my energy levels steadily improved- but best of all it made me smile, laugh, and now I look forward to doing it!"*
>
> **Lisa, TAS resident living with long COVID**

Symptoms and management tips

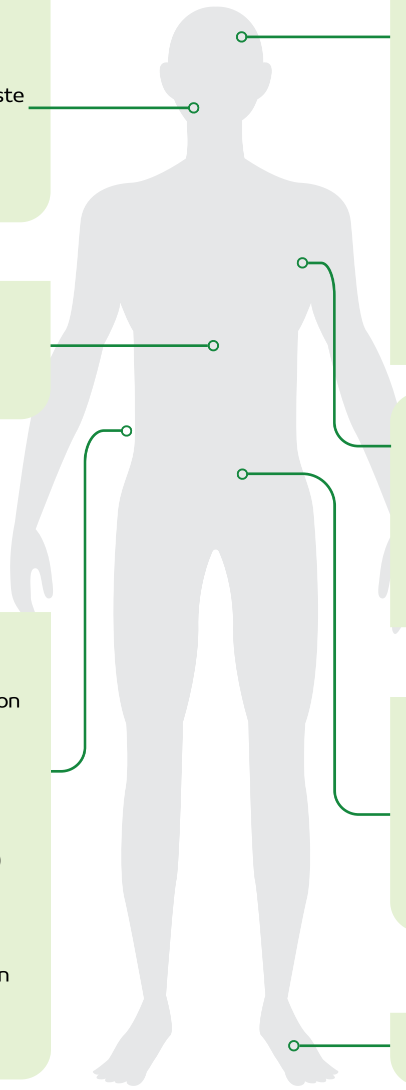

- Ringing in ears
- Poor hearing
- Loss of smell or taste
- Sore throat
- Altered voice
- Painful swallowing

- Stomach pain
- Nausea
- Loss of appetite
- Heartburn

- Poor sleep
- Anxiety
- Low mood/depression
- Fatigue
- Tiredness
- Lethargy
- Post exertional symptom exacerbation (PESE)
- Pins and needles
- Rashes
- Muscle weakness
- Joint and muscle pain
- Blood clots
- Skin changes

- Headaches
- Difficulty concentrating
- Poor thought processing
- Fatigue
- Memory loss
- Brain fog
- Dizziness and balance issues
- Postural Orthostatic Tachycardia Syndrome (POTS)
- Sensitivity to light and noise
- Hair loss
- Vertigo or lightheadedness

- Breathlessness
- Painful breathing
- Chest pain
- Back pain
- Cough
- Wheezing
- Palpitations

- Changes to menstrual symptoms
- Erectile dysfunction
- Changes in bladder or bowel habits
- Abdominal cramps or pain

- Nail or toe colour change

The following management tips are general in nature. Each person's symptoms and situation will differ, and you should talk to a healthcare professional to understand how to best manage your individual condition. For more information about treatment and investigations see the **getting help for long COVID section**.

With long COVID, your symptoms may frequently fluctuate and become worse after a period of improvement. It may be that there are moments of stress or increased physical or mental demands that cause these "fluctuations". When this happens, it is important to remember that overall, you are on a trajectory of improvement. The episodic nature of long COVID is explained in the long COVID video series on the website **longcovid.physio**.

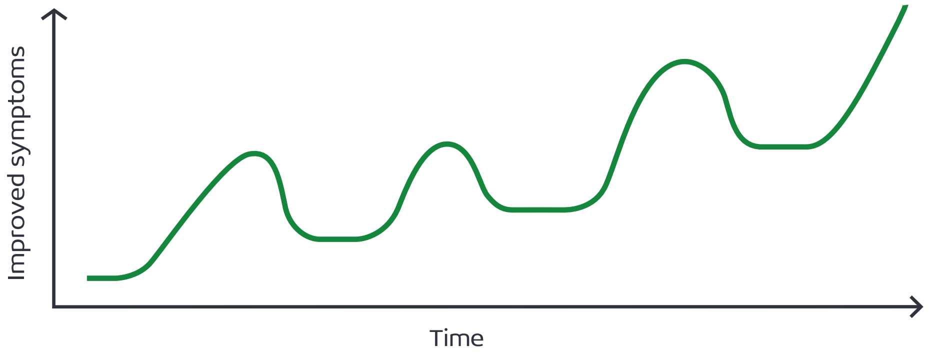

You should seek urgent medical care by calling the ambulance on triple zero (000) if you experience:

- Sudden chest pain or tightness
- Severe shortness of breath or difficulty breathing
- Confusion or seizure
- Fainting or collapse
- Rapid heart rate that does not settle with rest
- Worsening anxiety or mood, or if you have thoughts of harming yourself.

Fatigue

Fatigue is a very common symptom for people with long COVID. This type of fatigue is not just feeling tired after having a busy week or doing intense exercise, it is extreme exhaustion and lethargy. For people living with long COVID, fatigue may be a constant symptom, resulting in not being able to do everyday activities, or it may be variable, fluctuating after times of high stress or when doing activities with high energy demands.

The first step in managing fatigue is acknowledging it is real and that it significantly impacts your health and wellbeing. Other tips for managing fatigue include:

- Use **pacing** to break activities down into smaller, more manageable tasks.
- Establish good **sleep routines.**
- Try **relaxation techniques** such as meditation or having a relaxing bath.
- Eat **nutritious food** including a range of vegetables and fruits, lean protein and grains.
- After checking in with your healthcare professional, try some **light physical activity** such as yoga or tai chi for brief periods at first.

See sections **getting help for long COVID** and **talking to family and friends** for tips on communicating how fatigue is affecting you.

> *"I used to bounce out of bed in the mornings, but with long COVID, I wake up exhausted, and no amount of sleep seems to make any difference; even thinking about getting out of bed causes me anguish, but of course I do drag myself out of bed each morning... and everything is related. For example, my fatigue affects my lifestyle choices – if I'm exhausted then I don't have enough energy to cook a healthy meal and will end up having bickies and cheese."*
>
> **Elizabeth, SA resident living with long COVID.**

Post-Exertional Symptom Exacerbation (PESE)

Some people with long COVID experience Post-Exertional Symptom Exacerbation (PESE), also known as post-exertional malaise (PEM). PESE is different to fatigue. You may have the energy to do something, but then experience negative effects around 12 to 72 hours after the activity. For example, you may do the grocery shopping and feel fine at the time, but then a day later experience a flare up of your long COVID symptoms. Often this flare up will be very disabling and you may not be able to undertake your daily activities or feel like you can get out of bed.

> Many traditional rehabilitation programs are not suitable for patients experiencing PESE. If you suspect that you may have PESE it is important to talk to your healthcare professional to receive tailored advice on managing your symptoms.

Energy conservation is an effective way to manage PESE. It involves recognising what activities are making you tired. A good way to do this is by using a diary for a few weeks to keep track of what you did and how it affected you, and then look at patterns (e.g., I experience an increase in symptoms during the day if I go for a walk in the morning, but if I go for a walk at night, I don't have the same issues).

> Although doing a symptom diary can be helpful in the early stages of managing your PESE, keep it simple and keep in mind that tracking your activity and filling one out may be adding to your overall levels of fatigue. Once you know your patterns and triggers it's good to manage these without tracking if possible.

Once you know what activities are causing you to fatigue more quickly, you can map out a plan to best use your available energy each day. A good method for doing this is by using the **Three Ps: Pace, Plan and Prioritise**.

Pace	Plan	Prioritise
Pacing means taking breaks in between activities, and not doing anything strenuous for an extended period. A good way to do this is by breaking up your daily activities into small and manageable tasks. Try to pace yourself to avoid pushing your body to exhaustion.	To be able to pace well, effective planning is needed. Try to plan your day or week in advance and break up the activities that require more energy into smaller tasks.	It is important to ensure your essential tasks are planned for, and only then add in any other tasks. If you don't have enough time to rest in between tasks, maybe move something to the next week or consider removing it from your plans altogether.

Using the Three Ps will help to ensure you don't expend too much energy at once. For example, instead of spending a whole day cleaning the kitchen, first clean the sink and wipe the benchtops, then have a rest before emptying the dishwasher or wiping down the stove.

These diagrams show how pacing can work throughout the course of a day. The dotted COVID energy threshold line is the energy level that you are aiming to stay below, if possible. This is unique to you and will require some trial and error to understand your threshold. The pacing line shows how, with pacing, your energy exertion will go up and down over the course of a day, but ideally always stay under the COVID energy threshold. The not pacing example shows how if you expend energy above your long COVID symptom threshold it will lead to PESE.

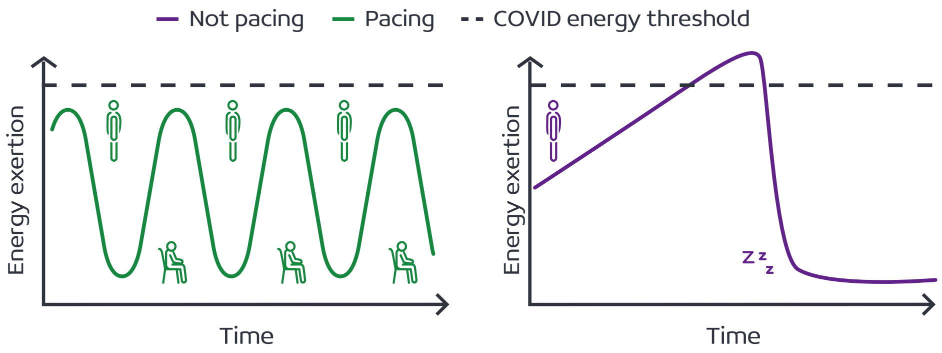

There are apps available to help monitor symptoms and plan activities. Keep in mind that using these apps too much may add to your mental load and lead to further fatigue. Try to only check in with them once per day to keep track. Once you have a good idea of what triggers your fatigue, it may be a good idea to stop using them altogether.

Remember that many things may contribute to your fatigue and Post-Exertional Symptom Exacerbations (PESE).

Some examples include:

Physical fatigue

Physical fatigue arises after doing physical activity and can result in muscle soreness and exhaustion.

Mental and cognitive fatigue

The most common sign of mental and cognitive fatigue is when you can't process information or concentrate as usual (often referred to as "brain fog"). This may include not being able to find words or forgetting things. It's important to remember that even things you may not consider to be tiring, such as reading the paper or looking at your phone, may be contributing to your fatigue.

Social fatigue

Even though it is important to maintain relationships with your friends and family, you may find spending time socialising can cause you to feel fatigued. You may want to consider meeting friends in a place where you can sit comfortably and let them know when you need to get some rest. See **talking to family and friends** for some more tips on explaining how long COVID is affecting you.

Breathlessness

Breathlessness is a common symptom that many people experience after having COVID-19. This is often because of the way that the virus affects your lungs, airways and respiratory muscles. If you are less active because you are unwell it may cause your muscles to become weak and in turn your breathlessness to get worse. The good news is that this negative cycle can be turned into a positive one, and by doing some activity your muscles will regain their strength and your breathlessness should improve.

All people with ongoing breathlessness should be evaluated by a healthcare professional. This is particularly important if the breathlessness is present at rest or with minimal activity, or if it suddenly worsens. People who are older or have underlying medical conditions such as asthma, Chronic Obstructive Pulmonary Disease (COPD) or heart disease should be evaluated by a doctor if they experience new or different breathlessness.

If no specific cause for your breathlessness is found your GP may refer you to a respiratory physician for further assessment and management.

It is helpful to learn practical ways to manage your breathlessness such as breathing control exercises. Some people may benefit from a structured **pulmonary rehabilitation program** to ease symptoms and improve their ability to exercise. Discuss with your GP whether a pulmonary rehabilitation program may be helpful for you and find out more by visiting lungfoundation.com.au.

If you have a pre-existing lung condition such as asthma or COPD having a written action plan can ensure you have optimal use of your medications to manage your condition. A written action plan, developed with your GP or respiratory healthcare professional can help to guide your management. You can download a **COPD action plan template** from Lung Foundation Australia's website or an **asthma action plan template** from Asthma Australia's website to take to your next appointment.

Breathing control exercises

Prior to having COVID-19 you may not have been as aware of your breathing as you are now. The body's normal reaction when breathlessness occurs is to breathe faster and shallower than usual. However, this is not an effective way to gain control of your breathing. If you are experiencing breathlessness or feeling like you are working harder to breathe than usual it may take up a lot of your energy. Thankfully, there are breathing techniques that can help you feel more in control, manage the uncomfortable feeling of breathlessness and improve your energy levels.

Breathing control promotes efficient breathing patterns and deters rapid breathing. Here are some different breathing techniques that can enhance your breathing.

Rectangular breathing

- Watch your stomach rise as you breathe in
- Relax your stomach as you let yourself breathe out
- Concentrate on the breath out, the in breath will take care of itself.

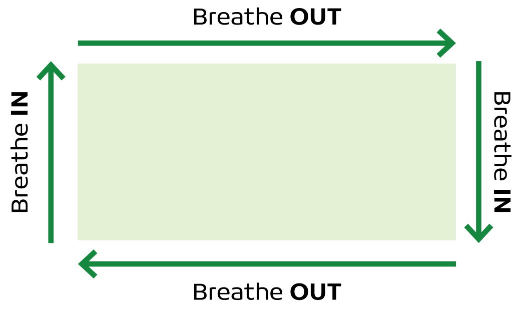

You can visualise this technique by imagining yourself breathing around a rectangle. Focus on taking long, slow breaths whilst letting the air completely out followed by a short breath in.

Paced breathing

This breathing technique is where you use the rhythm of your footsteps to pace your breathing. This can be done from a seated position. You may have one step to breathe in and a couple to breathe out, for example:

1. Breathe in on your left foot
2. Take a couple of steps
3. Breathe out on your right foot

Try using a handheld fan

Many people find using a battery-operated fan can help control breathlessness, and research has shown that a cool draft of air from a hand-held fan can be very effective. It works by circulating cool air over the face which helps to reduce breathlessness. Visit **lungfoundation.com.au** to see an instructional video of how to use a handheld fan effectively.

Cough

Having a persistent cough can be very debilitating and distressing. It can affect your daily activities, and you may feel more tired and experience increased pain and breathlessness, anxiety and difficulty sleeping. A cough can vary from person to person and may be dry or moist. For more information on chronic cough, visit **lungfoundation.com.au/chroniccough**.

Cough symptoms should be investigated with your healthcare professional.

Pre-existing conditions

The virus that causes long COVID typically causes inflammation within the airways. This inflammation has been shown to last for many months and can result in worsening of pre-existing airway problems that will worsen cough symptoms. Treatment of these conditions may help to improve your cough. If you have hayfever or sinusitis, asthma, COPD, another chronic lung disease or reflux, speak to your healthcare professional to see if your treatment is optimal.

Practical tips to reduce coughing:

- Keep your throat well hydrated, by regularly sipping water
- Avoid caffeine or alcohol that can dry your throat
- Try and breathe through your nose, if possible
- Avoid speaking for long periods or take breaks to reduce throat irritation
- If you need to cough but don't have a drink nearby, try swallowing repeatedly instead, this mimics the act of sipping water
- Smoking or vaping will worsen any cough, stopping will help to reduce your cough.

Excessive and chronic coughing may also change the quality of your voice.

If your cough is not improving over time, your GP may be able to refer you to a speech pathologist. Speech pathologists can help in the management of symptoms such as chronic cough, hoarse voice, difficulty swallowing or word finding difficulties.

Brain fog

The term "brain fog" refers to the feeling of having impaired thought processing, as if your brain is shrouded in fog. This makes it difficult for you to process information or to concentrate as you usually would. This may include struggling to plan your day or solve problems, remembering recent events and having difficulty finding words. These "cognitive impairments" will often make it much harder for you to perform your usual work, study or daily activities. If you are having these difficulties, it is important to communicate this with your family, friends, and/or employer to ensure they understand how brain fog is affecting you and how it may be affecting your work or study.

There are some strategies which may help ease your symptoms.

1. **Changes which can help improve memory**
   Manage your fatigue: often when you are fatigued other symptoms such as brain fog are worsened. When you plan your day, you may want to schedule in your thinking tasks for when you have the most energy, such as first thing in the morning. **Reduce anxiety levels, minimise alcohol intake,** and **maintain a healthy diet.**

2. **Adopt short-term memory strategies**
   Reduce distractions: if possible, try to do your work or study in a quiet place, with minimal distractions such as music, other people or loud noises.
   Slow down: if possible, try to do one task at a time to avoid making mistakes.
   Use simple communication: when you are unable to find the exact word or lose your train of thought in a conversation, keep it simple and find a different way to say it. Visualise and describe the object or idea, this can help you in finding the word quicker. If you do lose your train of thought in a conversation, don't be embarrassed to ask for a prompt from the person you're speaking with.

3. **Use external memory aids**
   Take notes: using lists or a diary to keep track can be useful when your memory is less reliable. Use your phone to **set reminders** to remind you of upcoming activities.

Cardiac (heart) symptoms

Cardiac symptoms such as palpitations and chest pain are common symptoms of long COVID. Often these symptoms are not a sign of serious disease, however the best course of action to take if you are experiencing them is to talk to your healthcare professional.

Palpitations

Heart palpitations are when you become aware of your heart beating. This can happen when your heart starts beating faster or harder. In some cases, people may have a feeling of their heart missing beats or "fluttering." Palpitations can make you feel anxious, but they are usually not serious and can happen to anyone. Some of the common causes could include being worried about something, consuming caffeinated drinks such as coffee, energy drinks, cola, tea, alcohol, strenuous exercise or being unwell (such as when you have a cold). If your palpitations do not last very long or can be linked to a cause such as those listed above, you will not usually need to do anything. You should see a healthcare professional if you have new palpitations, or if your palpitations do not get better, seem to last a very long time, are more frequent than usual, or are worrying you.

Chest pain

There are many things that can cause chest pain, and some are more concerning than others. For example, **chest discomfort** may be the result of coughing, reflux or muscle or joint problems. In the case of muscle or joint problems, the area is usually tender to touch, and you might notice it feels worse when you move or stretch the area.

Another type of chest pain is called **pleuritic pain**. Pleuritic pain is sharp and occurs when you take a deep breath in. **Angina** is a chest pain that occurs when your heart doesn't get enough blood or oxygen. Angina symptoms can be variable and may present as chest tightness or heaviness rather than pain. Some people also experience pain in other areas such as their arm, neck, jaw or even between their shoulder blades.

It can be hard to know whether your chest pain is related to a heart or lung problem (or even **heartburn**) so if you are experiencing chest pain, it is important to discuss this with your healthcare professional.

You should seek urgent medical care by calling the ambulance on triple zero (000) if you:

- Have palpitations while also experiencing other symptoms such as shortness of breath, chest pain or dizziness.
- Have chest pains that last a long time, or you are worried you might be having a heart attack.

Postural Orthostatic Tachycardia Syndrome (POTS)

POTS is a form of chronic orthostatic intolerance (inability to remain upright without symptoms) and dysautonomia (autonomic nervous system disorder). POTS is often triggered by viral infections (like COVID-19). If you have POTS, when you stand up or move into an upright position, you may experience an excessive increase in heart rate and debilitating symptoms such as dizziness, fainting, shortness of breath, chest pain, and lightheadedness.

Evidence on how to best manage POTS is varied, but the following may help your symptoms:

- See if you can do activities in a leaning or resting posture, rather than upright
- Increase your salt and magnesium intake
- Increase water intake to 2-3L per day
- Establish good **sleep routines**
- Try relaxation techniques such as meditation or yoga
- Engage in low grade **exercise** under direction of a healthcare professional.
- Identify and avoid triggers
- Try having small, frequent meals

For most people, it is unlikely that your POTS symptoms are a sign of serious cardiac disease, however you should talk to your healthcare professional to develop a tailored management plan. They will be able to prescribe medications which may help, if appropriate.

Sleep

Getting the right amount of good quality sleep is really important for feeling refreshed and energetic throughout the day. After being unwell, you may notice changes in your sleep patterns. This may include finding it difficult to fall asleep, waking up during the night and not being able to get back to sleep, or waking up and not feeling refreshed.

There are many reasons why long COVID may result in sleep disturbance. These include:

- Disruption to usual routines
- Altered circadian rhythm
- Coughing or pain symptoms disrupting sleep
- Sleeping during the day due to fatigue
- Change in lifestyle with less physical exercise and altered diet
- Greater stress and anxiety with ill-health
- Work/study interruptions.

Fatigue can also result in you feeling tired all the time, regardless of how much sleep you're getting. Here are some tips to help you sleep better and maintain sleep hygiene:

- Drink enough water during the day but avoid drinking liquids or eating food in the two hours prior to bed
- Try to get some sunlight first thing in the morning
- Light exercise such as yoga or tai chi during the morning or earlier in the day
- Try not to sleep during the day, but if you have to nap, limit to 20-30minutes
- If you lay down during the day, avoid your bed, so as to not associate your bed with simply lying down rather than sleeping
- A few hours before bedtime reduce your intake of sugary foods, caffeinated drinks and alcohol
- Smoking cigarettes or e-cigarettes/vapes will also disturb your sleep and should be avoided. See the **quitting smoking page** on Lung Foundation Australia's website for more information.

It may also be beneficial to develop a regular sleep routine that works for you to give you the best chance of getting a good night's sleep.

- Aim for 7-9 hours of sleep each night, and to go to bed and wake up around the same time every day
- Create a calm environment by turning the lights down, reading, listening to music, or listening to an audiobook before bedtime
- Keep the temperature in your room cool
- Avoid using anything with a screen before bedtime (for example mobile phone, tablet or computer screen).

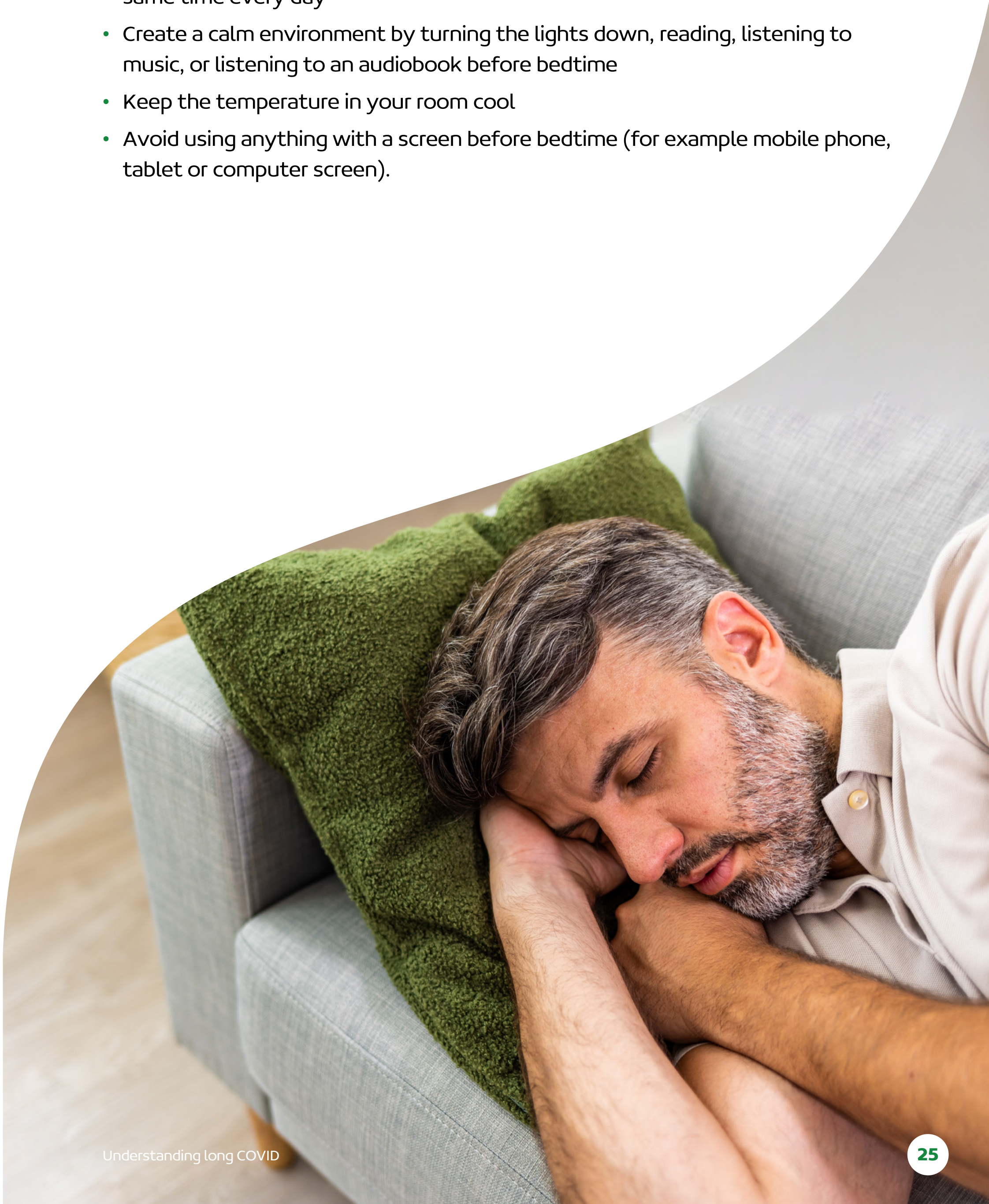

Emotional wellbeing

It's important to recognise that feeling flat and worrying about your health is a normal reaction to being diagnosed with something that many people don't yet understand. You may have frustrating conversations with people (possibly including healthcare professionals) who dismiss your symptoms, or struggle to adjust to a change in your routines. The emotional impact of having to combat social attitudes and stigma can be significant. Knowing this and focusing on your emotional wellness can be a very important component of managing your overall wellbeing.

Maintain perspective

When feeling anxious or worried about the current situation, it may help to remember healthcare professionals and researchers are working hard to help those in need and find effective treatments.

When you are feeling worried, it can cause physical changes to your body, such as:

- Increased heart rate
- Faster breathing
- Sweating
- Stomach pains
- Repetitive intrusive thoughts.

Looking after your emotional wellbeing

- When you are feeling anxious or stressed, you may find yourself breathing more quickly. While slow deep breathing techniques are useful for most people, they may not be relaxing if your long COVID symptoms include breathlessness. Lung Foundation Australia have a **series of videos and resources** available online to guide you through **mindfulness and self-compassion practices**.
- Language and thoughts are powerful contributors to emotions so try **to be aware of your own self-talk**. This involves recognising when you have unhelpful thoughts and being able to turn them into balanced thoughts. For example, if you have a thought along the lines of "I'll never get better", it can be helpful to reframe this thought to "many people with long COVID have found ways to manage their symptoms and I can too".
- If you wake up and feel low in energy, try to avoid telling yourself that you're having a bad day. Reframe it as a low energy morning and you may feel more up for an activity by the afternoon. Building this 'wriggle room' into self-talk opens the possibility for you to feel different later.

Even once you are effectively using techniques to manage your mental wellbeing, it is important to recognise that some days will be better than others. On the days when you feel more worried or stressed be sure to reach out to your support networks such as family and friends.

Seek support

It may also be beneficial for you to link in with a peer support network. Contact Lung Foundation Australia on freecall **1800 654 301** to find out more.

Beyond Blue also have specific resources on COVID-19.

If you need to talk to someone, reach out to a support service such as **Lifeline** on **13 11 14**, who provide 24-hour support to listen to how you are feeling and offer you information and advice.

> *"Once I accepted that it wasn't getting better by "tomorrow", it became easier to take each day as it comes, and my stress levels dropped dramatically. Then I could find some pleasure in the day, and stopped defining myself by what I achieved in a day, by my work, by my old life, and realised that my worth isn't tied to those things- that just existing is still a valuable way to live."*
>
> **Lisa, TAS resident living with long COVID**

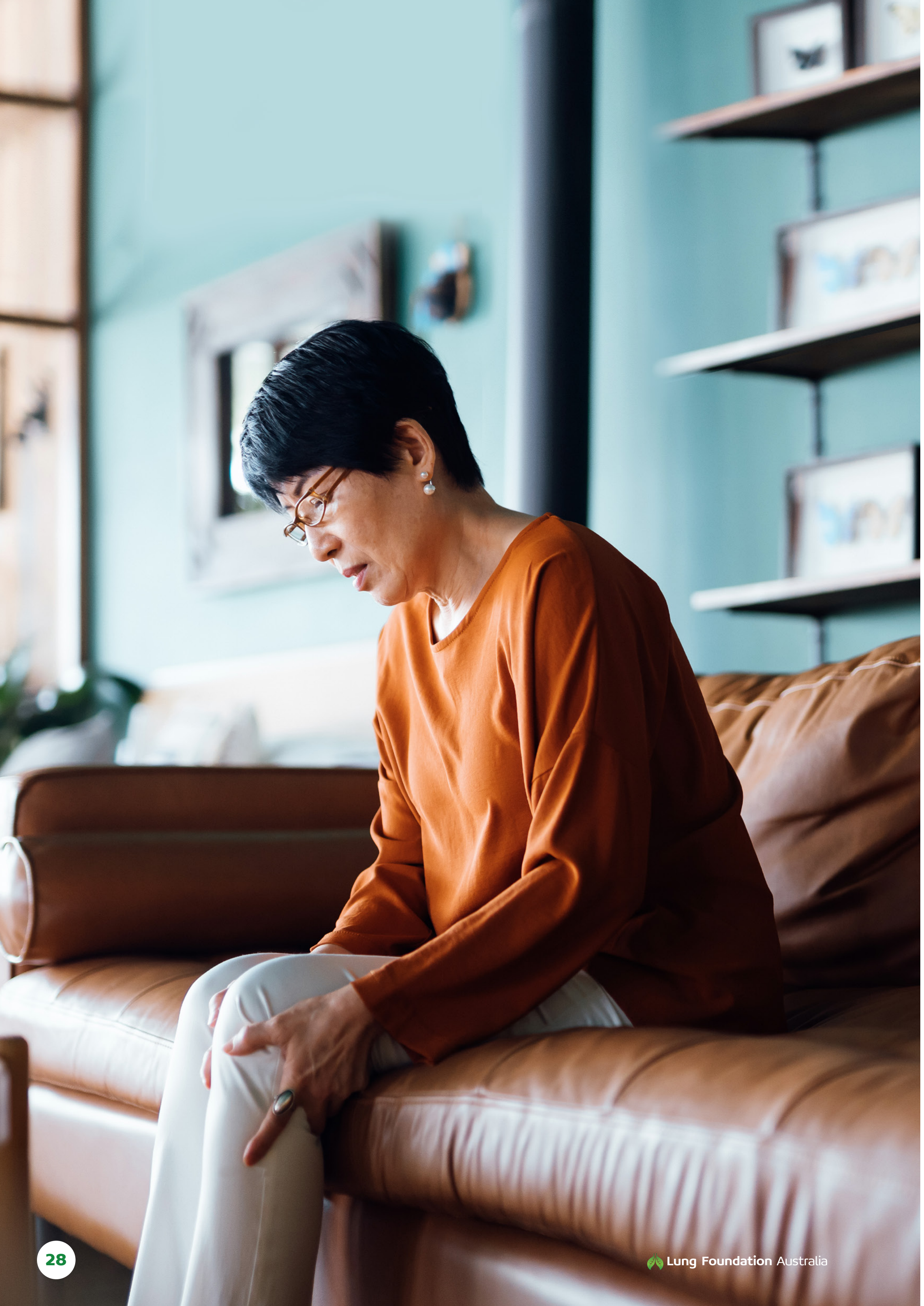

Pain

Long COVID is an inflammatory condition that may result in new pain in your joints or elsewhere in your body. This pain may be located in specific areas of your body (e.g., headaches or joint pain) or more generalised. The effects of feeling persistent pain can be exhausting and can affect your fatigue levels, mood, and ability to concentrate.

Pain can be managed in various ways, and it's always best to consult with your healthcare professional for their advice before undergoing any specific treatments. While waiting for a consultation, over the counter medications such as paracetamol or ibuprofen can be taken with food to help manage pain. Be sure to speak to your pharmacist and read the packet of these medicines to avoid exceeding the recommended dose per day or taking them regularly for too long.

Quality sleep, **healthy lifestyle choices** and **relaxation techniques** may also aid in pain management.

Gastrointestinal symptoms

Gastrointestinal symptoms are fairly common after having an acute COVID-19 infection. This includes symptoms such as heartburn, constipation, diarrhoea, and abdominal pain. Many people report these symptoms improving over time. Sometimes these symptoms may be linked to mental health symptoms such as anxiety. See the **worries and mental wellbeing** section for some tips for managing worries.

If you are concerned about your gastrointestinal symptoms, it is important to discuss this with your healthcare professional. In the meantime, you may want to try the following self-management strategies:

- Avoid foods that trigger your symptoms
- Introduce more foods that are high in fibre
- Drink plenty of water
- Aim to get quality **sleep**
- Eat and drink in an upright and seated position
- Consider taking medicine or other treatments to help with your symptoms.

Other tips

Returning to exercise

Before returning to exercise, it is important to talk to a healthcare professional. Everyone's recovery looks different, and having a tailored exercise program will help to ensure that you don't over or under exercise. It's likely that you will have to reintroduce exercise into your weekly plans slowly, but gradually you should be able to build your capacity again. See the **pacing** section for more information to help you manage this. Lung Foundation Australia have a **maintaining movement video series** which may be helpful.

Maintaining a healthy diet

Some long COVID symptoms, such as fatigue, may make it harder to ensure you keep a healthy diet and stay well hydrated. Maintaining a healthy diet, which includes a variety of food groups, is essential for good health. If needed, you may want to discuss your diet with a healthcare professional such as a dietitian or your GP. Using pre-prepared frozen meals or asking friends or family to cook a meal for you can be helpful to save energy. When you do meal preparation, it may be useful to sit down to conserve energy.

Stopping smoking and vaping

Smoking and vaping causes harm to your immune system and lungs. If you are recovering from a virus such as COVID-19, smoking and vaping will slow down your recovery and increase your risk of reinfection. There are other significant benefits of quitting smoking and vaping, such as increasing your life expectancy and reducing the risk of diabetes or heart and lung disease. Speak to your doctor about treatment options to help you quit smoking and vaping, such as nicotine replacement therapy (NRT), other oral medications and support such as coaching and counselling. See the **quitting smoking page** on Lung Foundation Australia's website for more information.

Reducing alcohol consumption

Alcohol use weakens the immune system and may hinder your body's ability to improve your long COVID symptoms. Drinking alcohol may also worsen long COVID symptoms so it's important to try to reduce your consumption.

Returning to work

Many long COVID symptoms may affect your ability to work as usual. It's important to work with your employer to best manage your workload and your symptoms to ensure that work is not causing you to become more unwell or delaying your recovery.

Some things to discuss with your employer may include:

- A phased return to work – meaning that you will have a gradual increase of hours and responsibilities over an agreed upon period of time
- A flexible working arrangement – e.g., reducing your hours, or doing your hours flexibly across the week when you have more energy, or working from home, if possible, to conserve energy
- If you are unable to meet the requirements of your role, discuss options for changing roles within your organisation
- Review your work responsibilities and possibly delegate some tasks to other members of your team.

It's important to acknowledge that returning to work after being unwell may be distressing.

> If returning to work is causing you to worry, it may be beneficial to talk this through with your healthcare professional to get tailored advice specific to your situation.

Returning to school

Navigating a chronic health condition can impact a child's school experience. If your child has been unable to attend school due to being unwell, they may need additional support. For more information on the types of support available for students, see Lung Foundation Australia's chronic conditions and schooling fact sheet via lungfoundation.com.au.

Level 2, 11 Finchley Street, Milton, QLD 4064
PO Box 1949, Milton, QLD 4064
enquiries@lungfoundation.com.au

1800 654 301

REGISTERED CHARITY
acnc.gov.au/charityregister

©Lung Foundation Australia.©Lung Foundation Australia. BOOK0523V1LONGCOVID
